# Comment on: “Preventing chemotherapy-induced alopecia: a prospective clinical trial on the efficacy and safety of a scalp-cooling system in early breast cancer patients treated with anthracyclines”

**DOI:** 10.1038/s41416-019-0576-5

**Published:** 2019-09-17

**Authors:** Jun Kako, Kohei Kajiwara, Masamitsu Kobayashi, Yasufumi Oosono

**Affiliations:** 1 Division of Nursing Science, Graduate School of Biomedical and Health Sciences, Hiroshima University, Minami-ku, Hiroshima, 734-8553 Japan; 2 Faculty of Nursing, National Defense Medical College, Saitama, 359-8513 Japan; 3 Community Health Nursing, National Defense Medical College, Saitama, 359-8513 Japan

**Keywords:** Oncology, Cancer

We have read, with great interest, the recent paper titled “Preventing chemotherapy-induced alopecia: a prospective clinical trial on the efficacy and safety of a scalp-cooling system in early breast cancer patients treated with anthracyclines” by Munzone et al.,^1^ which evaluated the effect of a scalp-cooling system on chemotherapy-induced alopecia (CIA). We would like to highlight additional points of discussion regarding this study.

The authors evaluated the concordance between patient and nurse/physician Dean’s score and reported that Cohen’s kappa was 0.41, indicating moderate agreement; however, 53.8% of patients evaluated their hair loss at ≤50%, whereas 79.8% of the medical staff evaluated patient hair loss at ≤50%. There was a significant difference between the patient and medical staff evaluations (*p* < .001). This indicates that the medical staff underestimated the CIA. The report of moderate agreement is encouraging; however, we believe that the authors should have been mindful of the potential for missing patient distress.

The authors reported on breast-related quality of life among the patients starting chemotherapy with scalp cooling; however, we believe that some background-related data are lacking. For instance, the success rate, failure rate and Dean’s score could have been reported for the 58 patients who completed the European Organization for Research and Treatment-QOL questionnaire and breast cancer specific module questionnaires. In addition, was there a statistically significant difference regarding body image between the success and failure groups?

We believe that addressing the questions raised would provide a clearer picture of the results of the study by Munzone et al.

## Data Availability

Not Applicable.

## References

[1] Munzone E., Bagnardi V., Campennì G., Mazzocco K., Pagan E., Tramacere A. et al. Preventing chemotherapy-induced alopecia: a prospective clinical trial on the efficacy and safety of a scalp-cooling system in early breast cancer patients treated with anthracyclines. *Br. J. Cancer*10.1038/s41416-019-0520-8 (2019). 10.1038/s41416-019-0520-8 PMC6738323 31303642

